# Supplementary material for: Exposure to stereotype-relevant stories shapes children’s implicit gender stereotypes
Source: PLoS One. 2022 Aug 3;17(8):e0271396. doi: 10.1371/journal.pone.0271396 (PMC9348658; doi:10.1371/journal.pone.0271396)
Supplement: S1 File — Additional detail on methods and supplementary analyses. (DOCX) [file pone.0271396.s001.docx]

Supporting Information for:

Exposure to Stereotype-Relevant Stories Shapes Children’s Implicit Gender Stereotypes

**Table of Contents**

| **Section: Method** | | |
| --- | --- | --- |
|  | Full Vignettes | p. 03 |
|  | Full List of Measures | p. 11 |
| **Section: Results** | | |
|  | Manipulation Check Results | p. 12 |
|  | Perceptions of Tennis/Swimming | p. 12 |
|  | Condition by Continuous Age Interaction on Implicit Gender Stereotypes |  |
|  | Gender by Condition by Age Group effects on Self-Concept | p. 13 |
|  | Implicit Bias Predicting Self-Concept Controlling for Condition | p. 16 |

**Full Vignettes**

**Stereotypical Condition: Story 1***Note, we hide pictures of children in these examples to ensure privacy.*


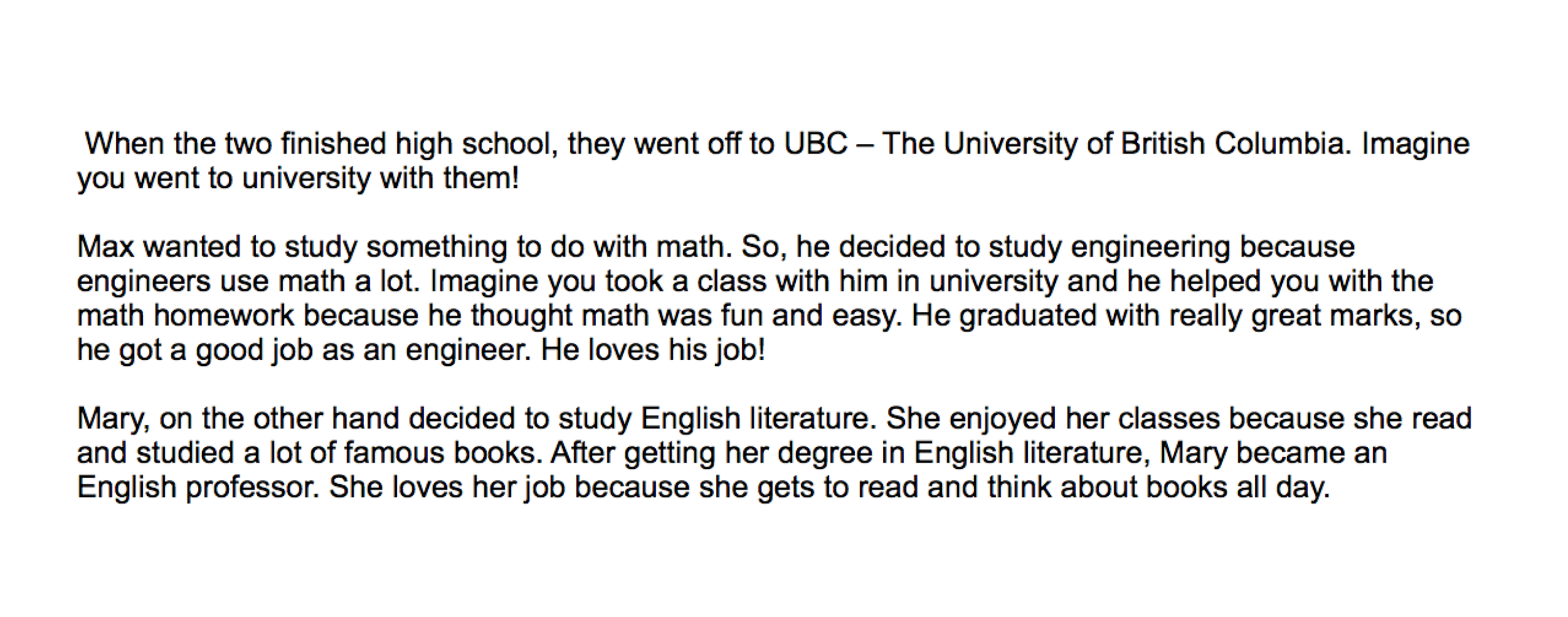
**
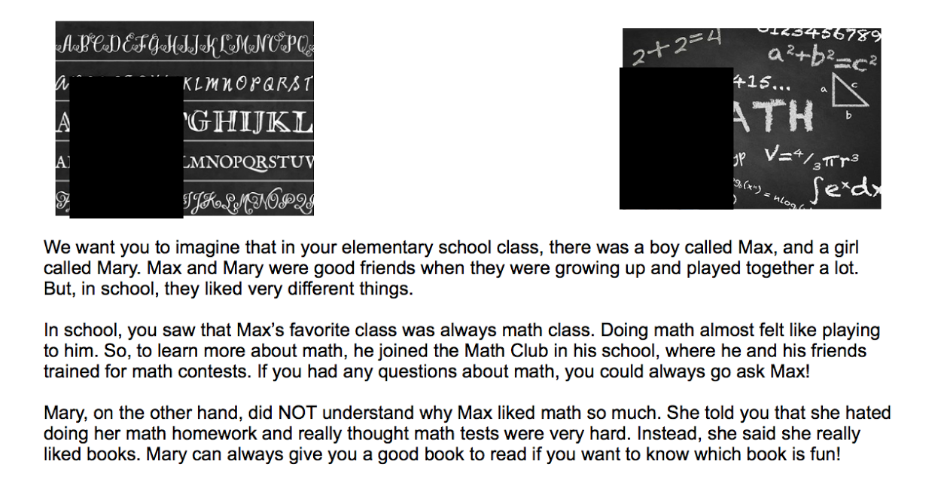
**

**Manipulation Check**

- In the story you heard, who liked math more?
- In the story you heard, who liked reading and writing more?
- Who became an engineer when they grew up?
- Who became an English professor when they grew up?

**Stereotypical Condition: Story 2**


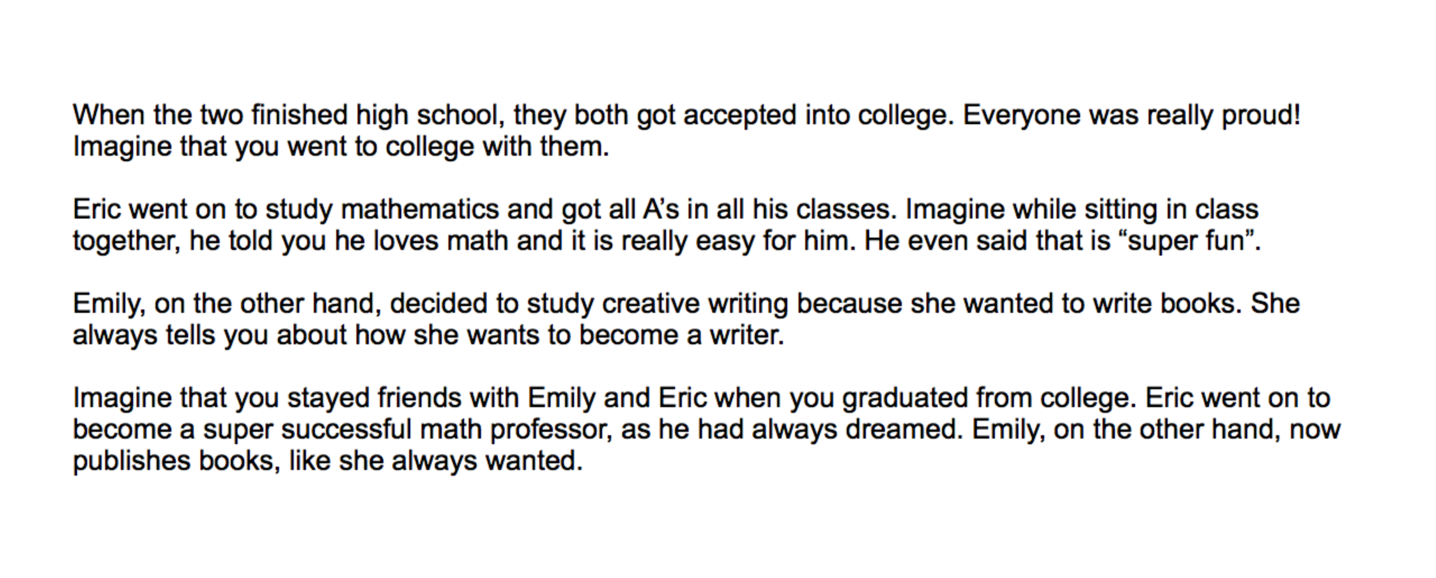
**
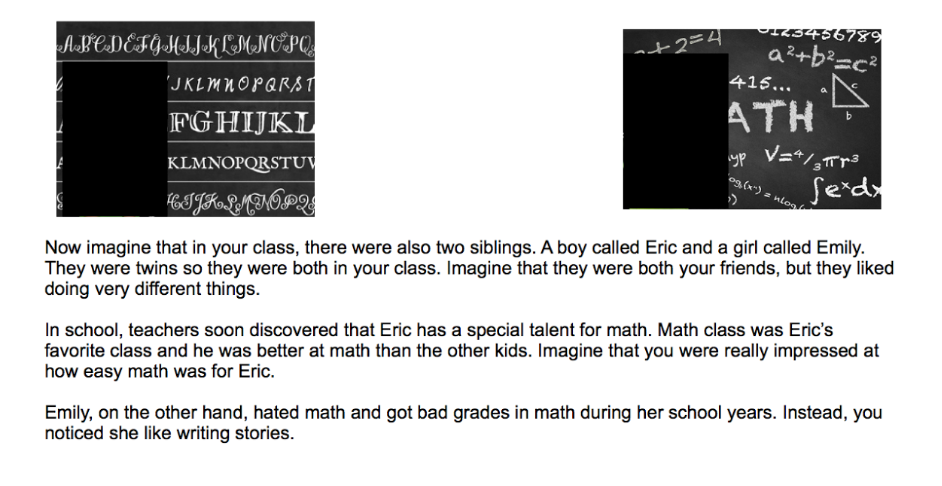
**

**Manipulation Check**

- In the story you heard, who liked math more?
- In the story you heard, who liked reading and writing more?
- Who became a math professor when they grew up?
- Who became a writer when they grew up?

**Control Condition: Story 1

Note Control Stories were also counter-balanced for which gender preferred which sport.**


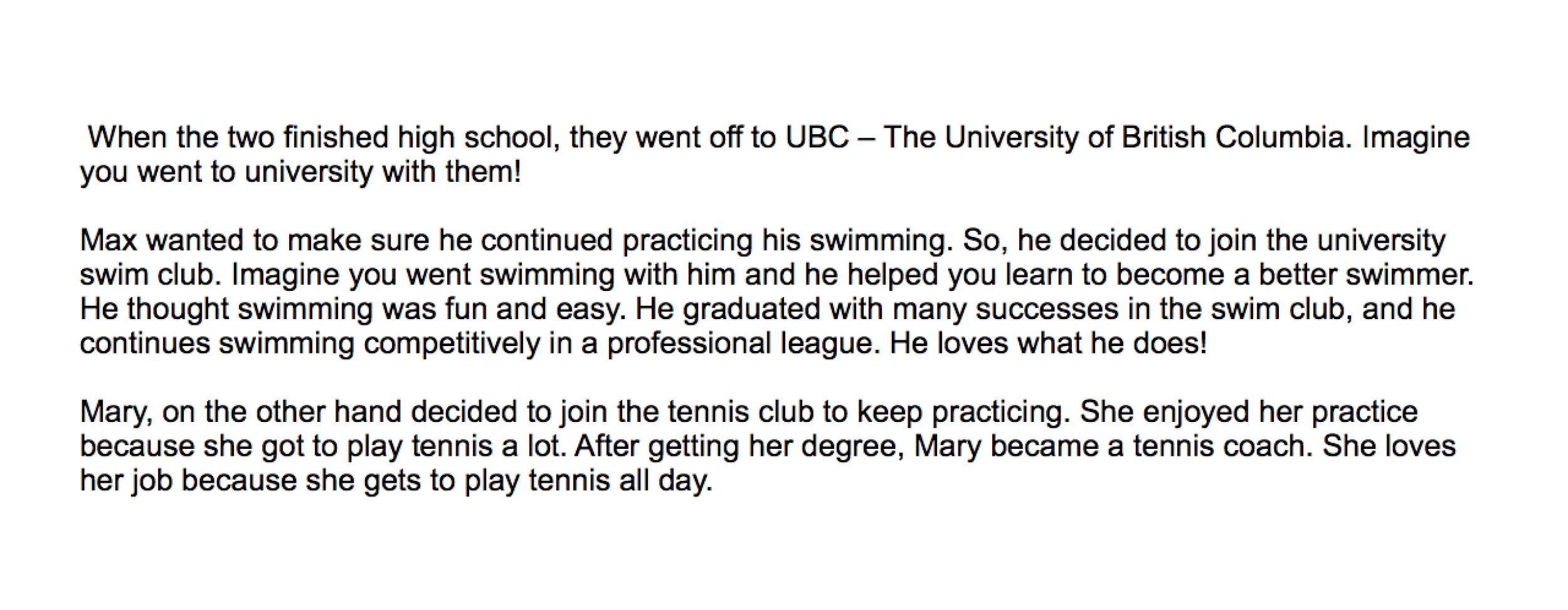
**
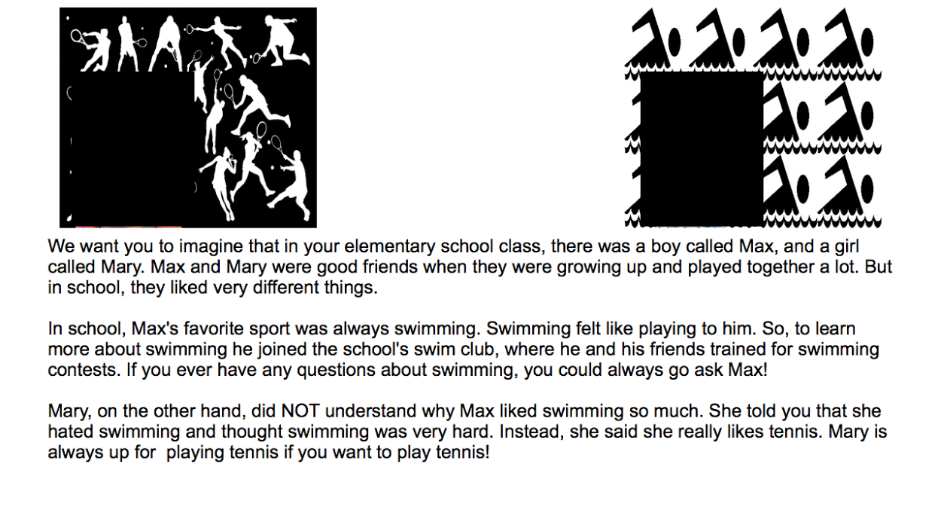
**

**Manipulation Check**

- In the story you just heard, who liked swimming more?
- In the story you just heard, who liked tennis more?
- Who became a swimmer when they grew up?
- Who became a tennis player when they grew up?


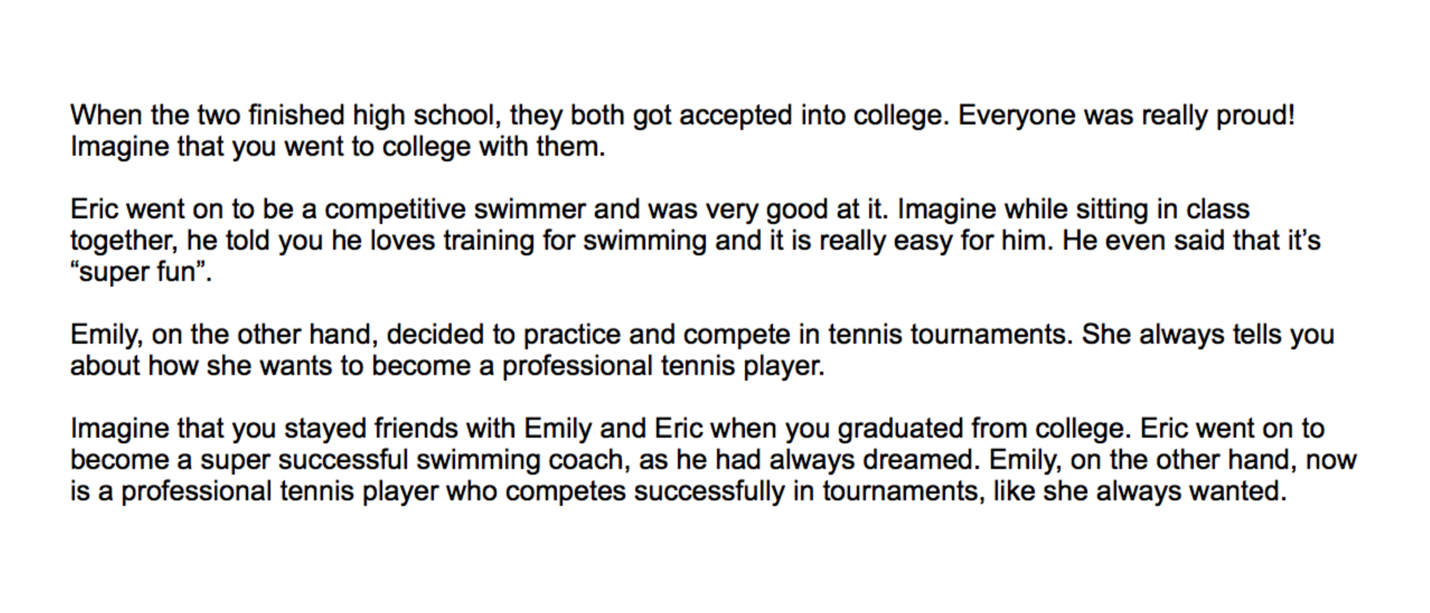
**Control Condition: Story 2

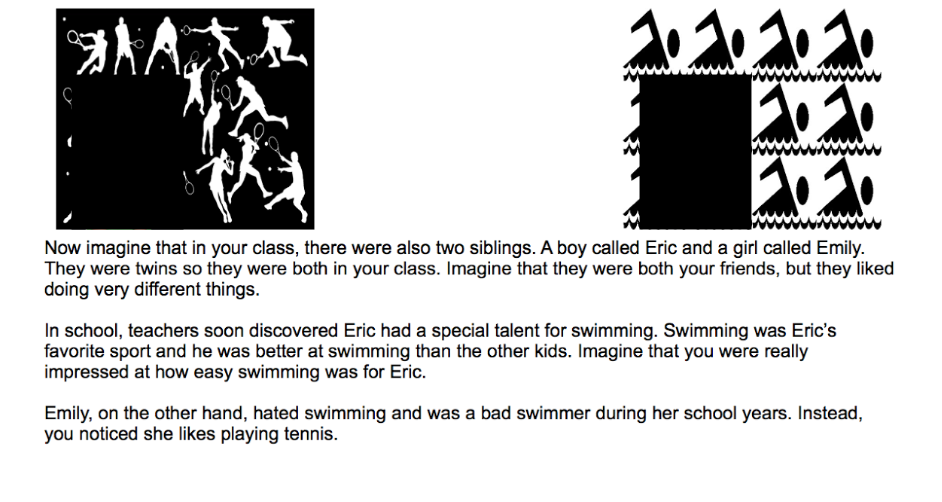
**

**Counter-stereotypical Condition: Story 1

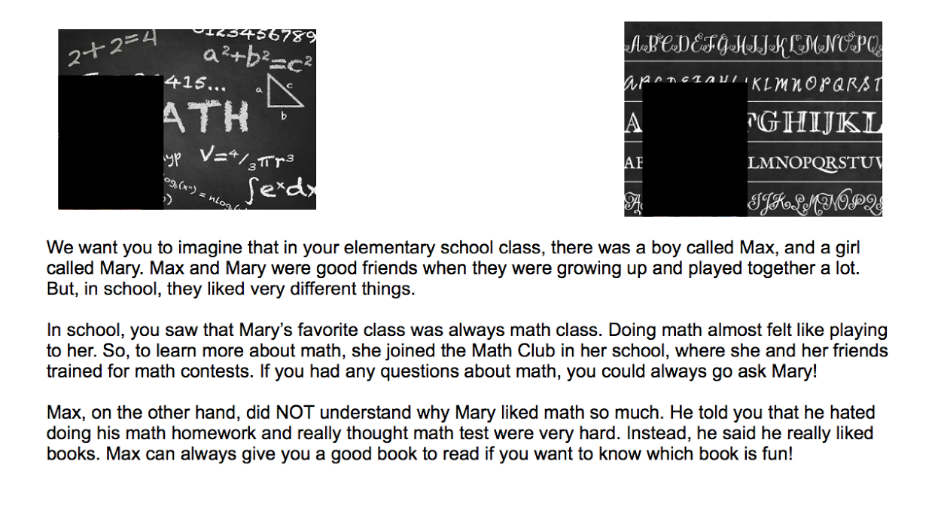
**
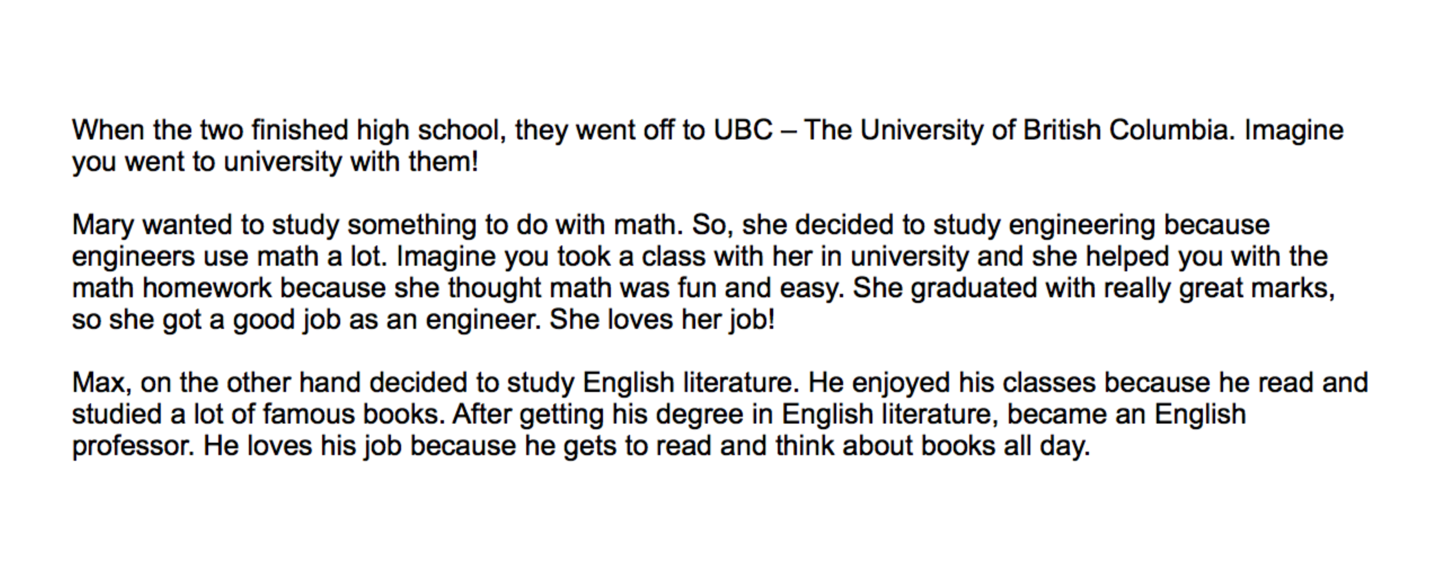


**Manipulation Check**

- In the story you heard, who liked math more?
- In the story you heard, who liked reading and writing more?
- Who became an engineer when they grew up?
- Who became an English professor when they grew up?

**Counter-stereotypical Condition: Story 2
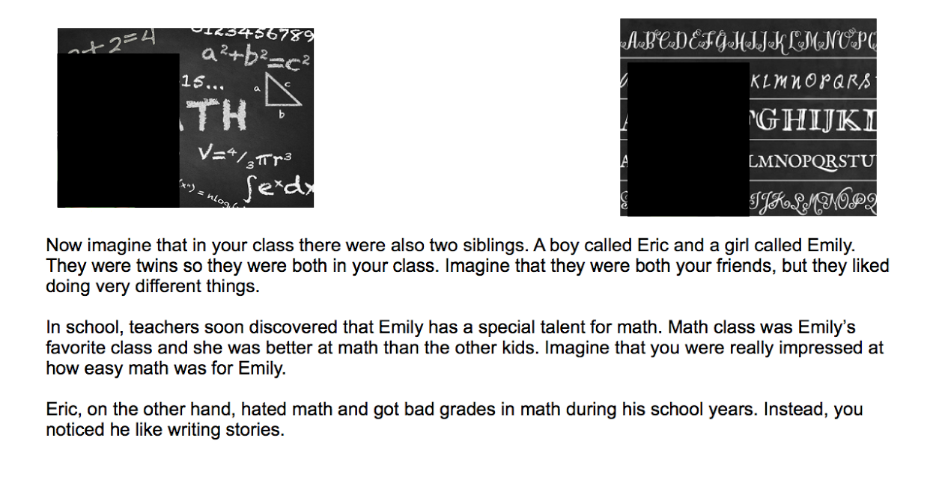
**


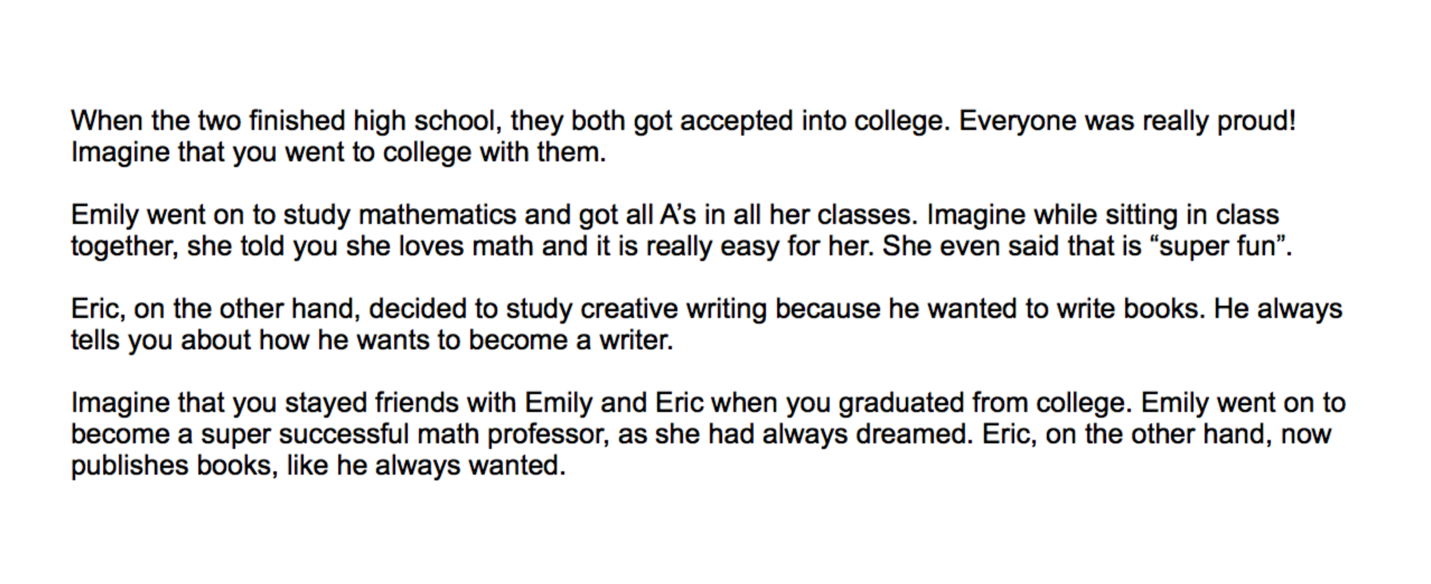


**Manipulation Check**

- In the story you heard, who liked math more?
- In the story you heard, who liked reading and writing more?
- Who became a math professor when they grew up?
- Who became a writer when they grew up?

**Full List of Measures**

**Child Measures**

- How old are you?
- Are you a boy or a girl?
- Do you have siblings?
- Implicit math-gender stereotype IAT
  - *Math* words: numbers, count, addition, math
  - *Reading* words: words, books, read, letters
- Implicit Math self-concept IAT
  - *Me* words: mine, myself, my, I
  - *Other* words: them, themself, their, they
- Explicit math self-concept
  - How good at math are you?
  - How good are you at learning new things about math?
  - How fun do you think it is to do math?
  - How much do you like to do math?

**Parental Questionnaire**

**Introduction.** As part of this study, we are interested in understanding children’s academic performance and how it relates to their implicit gender stereotypes about academic ability. The following questionnaire will ask you a number of questions about your child’s academic performance. These questions will require reflection on your own child’s academic performance as well as reflection on your child’s gendered behavior. Are you comfortable with filling out this questionnaire?

- Demographic Information
  - What is your child’s first name (please do not enter last name)?
  - What is your CHILD’S gender?
  - What is YOUR gender?
- Frequency of Science Center Visits:
  - How often has your child visited Science World in the past (either with you or someone else)?
  - How often has your child visited ANY science centres or science museums (INCLUDING visits to Science World) either with you or someone else?
- Perception of Child’s Math Ability:
  Please answer the following statements as they currently characterize your child’s academic performance. For each question, select the response that most accurately describes your child.
  - In comparison to other children, how difficult is math for your child?
  - In comparison to other children, how is your child’s performance in math?
  - How well do you think your child will do in math next year?
- Perception of Child’s Academic Ability
  - In comparison to other children, how difficult is school (in general) for your child?
- Degree of Masculinity and Femininity:
  - Compared to other children that are the same AGE and GENDER as your child, how masculine is your child?
  - Compared to other children that are the same AGE and GENDER as your child, how feminine is your child?

**Results**

Data and experiment files can be found under: https://osf.io/2h3xu/.

**Manipulation Check Results**

After each of the two vignettes, children were asked four questions to assess whether they remembered what they learned in the story. Correct responses were coded as 1 and incorrect responses were coded as 0. For each child, we then calculated the percent of correct responses out of 8. Analyses indicated that on average, children answered 98.17% of questions correctly, indicating that they were generally able to understand our stories. To examine whether children’s understanding of the stories differed markedly by age, gender, or condition, we conducted a gender x condition x age group analysis. This analysis indicated that there were no gender, *F*(1, 323) = 0.76, *p* = .383, or condition differences, *F*(1, 323) = 0.05, *p* = .947, in children’s answers to the manipulation checks. Results did reveal a main effect of age group, *F*(1, 323) = 8.63, *p* = .004, with older children (*M* = 99.33%, *SD* = 4.78%) answering slightly more questions correctly than younger children (*M* = 96.33%, *SD* = 9.25%). Taken together, these results suggest that even younger children in our sample, on average, had a good understanding of our vignettes.

**Perceptions of Tennis/Swimming**

To determine whether the sports we chose for our control conditions were not perceived to be strongly preferred by one gender over the other (and thereby cue gender differences), we measured whether children thought of these sports as equally liked by boys and girls. To this end, we collected a separate pilot sample of 47 children (24 boys/23 girls, mean age = 8.43). Four additional children were excluded from these analyses because they reported not being sure what either tennis or swimming were. We asked children about tennis and swimming, two questions each, in randomized order. First, children were asked “Do you know what tennis/swimming is?”, then children were asked “Who likes tennis/swimming?” and were given three different answer options: 1) boys like tennis/swimming more than girls, 2) both boys and girls like tennis/swimming, or 3) girls like tennis/swimming more than boys. For both tennis (*M* = 2.00, *SD* = 0.42) and swimming (*M* = 2.00, *SD* = 0.30) the mean answer indicated that children largely thought that these sports were liked equally by boys and girls. Indeed, children chose the gender equal option 82.98% for tennis and 91.49% for swimming. These results suggest that these sports are perceived as relatively gender-neutral by children and were thus unlikely to act as a salient prime of gender differences.

**Condition by Continuous Age Interaction on Implicit Gender Stereotypes**

Our key analyses examined whether effects of condition differed between younger vs. older children, treating age-group as a categorical variable. While it is theoretically sensible that reaching certain developmental milestones can fundamentally alter how gendered information is process (Halim et al., 2011), changes with age could also arguably be gradual and quantitative change rather than qualitative shift. In this case, it would be more sensible to analyze age as a continuous moderator of condition effects on implicit gender stereotypes. When we did such analyses, we found a very similar pattern to the key analyses reported in the main manuscript; a pattern suggestive of an interaction but with no statistically significant results.

More specifically, we conducted a regression analysis where implicit gender stereotypes were predicted from gender (as a control variable), continuous age (standardized), condition, and the continuous age by condition interaction. Since the condition has three levels, we dummy coded with the counter-stereotypical condition as a reference group (so that dummy codes quantify how the counter-stereotypical condition differed from the control and the stereotypical conditions).

Parallel to results with the categorical age variable, this analysis showed a significant main effect of age; older children showed stronger implicit stereotypes than younger children did, *ß* = .04, *SE*=.04, *t*(311) = 1.69, *p* = .031. Neither interaction terms for counter-stereotypical vs. control, b = -.05, *SE* = .05, *t*(311) = -0.93, *p* = .351, nor the interaction term for counter-stereotypical vs. stereotypical, b = -.09, *SE* = .05, *t*(311) = -1.69, *p* = .092, reached conventional statistical significance.

Even though we found no formal interact effect of age and condition, the interaction between age and the dummy code comparing the counter-stereotypical condition vs. the stereotypical condition was marginal (*p* = .092), suggesting that implicit gender stereotypes may differ more between these two conditions for younger compared to older children. These results are tentative but parallel our main manuscript results when we divide children into categories of older vs. younger. Decomposing this marginal interaction on a purely exploratory basis suggested that the contrast between the counter-stereotypical vs. stereotypical was non-significant for older children (+1SD on age), b = .09, *SE* = .07, *t*(311) = -1.27, *p* = .205, but statistically significant in younger children (- 1 SD on age), b = .27, *SE* = .07, *t*(311) = 3.62, *p* < .001.

**Condition by Gender and Age Group Analyses on Self-Concept**

To assess whether effects on self-concept differed markedly between younger and older children, we created a variable that split our sample below and above the median age of 8.80 years as an exploratory analysis, similar to the median split age in Gonzalez and colleagues’ analyses on racial attitudes [1]. We thus had a group of younger (*M*_age_ = 7.96, *SD* = 0.57, *n* = 167) and a group of older children (*M*_age_ = 9.79, *SD* = .60, *n* = 169).

To test for condition differences in boys and girls, we conducted a 2 (gender: boy vs. girls) x 3 (condition: stereotypical, control, or counter-stereotypical) x 2 (age-group: younger vs. older) analysis of variance with 1) implicit and 2) explicit math self-concept as dependent variable. There was only a marginal main effect of gender on explicit math self-concept, with girls (*M* = 3.51, *SD* = 0.92) reporting a marginally lower explicit math self-concept than boys (*M* = 3.71, *SD* = 0.922), *F*(1, 324) = 3.26, *p* = .072, *η*^2^ = .01. As can be seen in Figure S2, despite girls’ implicit math self-concept being descriptively higher in the counter-stereotypical vs. stereotypical condition (*d* = .27), results from these analyses revealed that there were no other main effects of gender, age, condition, or interactive effects, *F* < 1.80, *p* > .180, *η^2^* < .012, suggesting such effects on implicit math self-concept were absent or smaller than what we had power to detect^^[[1]](#footnote-1)^^.

**Self-concept effect by age.** As in the main analyses, gender by condition analyses for the two different age groups separately yielded no significant effects of gender, condition, or their interaction, *Fs* < 2.12, *p*s > .124, *η*^2^s < .03 in either younger or older children.

*Figure S2.* Implicit Math Self-concept by Age Group, Gender, and Condition

**Implicit Bias Predicting Self-Concept Controlling for Condition**

To ensure that the observed marginal interaction between gender and implicit stereotypes predicting implicit math self-concept was not spurious due to condition, we repeated our main analyses, now adding condition as a control variable. As in previous analyses, condition did not predict implicit math self-concept, *ß* = -.02, *SE* = .03, *t*(290) = -0.35, *p* = .729. As with analyses not controlling for condition, results showed that the expected gender by implicit stereotype interaction was marginally significant, *ß* = -.16, *SE* = .04, *t*(290) = -1.93, *p* = .054. Decomposing this interaction suggested that implicit stereotypes were still marginally predictive of a lower math self-concept for girls, *ß* = -.16, *SE* = .03, *t*(290) = -1.93, *p* = .055, but were not predictive for boys, *ß* = .07, *SE* = .03, *t*(290) = 0.79, *p* = .428*.* Thus, in our sample, we have only tenuous evidence that girls’ implicit stereotypes predicted their own self-association with math.

1. A sensitivity analysis using G*Power examined which effect size we were powered to detect with 80% power. Analyses focused on the difference between girls in the stereotypical vs. counter-stereotypical conditions. Results suggested that we had 80% power to detect an effect size of *d* = .55. [↑](#footnote-ref-1)
