# Supplementary material for: Exposure to stereotype-relevant stories shapes children’s implicit gender stereotypes
Source: PLoS One. 2022 Aug 3;17(8):e0271396. doi: 10.1371/journal.pone.0271396 (PMC9348658; doi:10.1371/journal.pone.0271396)
Supplement: Attachment — Submitted filename: ChildExemplarPaper_RevisionLetter.docx [file pone.0271396.s002.docx]

# Dear Dr. Shook,

My co-authors and I sincerely appreciate the time you and the reviewers invested in helping us to improve our manuscript [PONE-D-21-23166]. We have carefully taken into account every comment by the reviewers and have addressed each through corrections, improved writing, and/or additional analyses. In this process, my team and I believe we were able to substantially improve our work. This would not have been possible without the thoughtful contributions of our reviewers. Below, we detail each comment and describe what we did to address it.

In addition, the submission system does not allow us to make edits to the “Financial Disclosure” and “Competing Interest” sections in the portal. As requested, we include this information here. Competing interests should say “The authors have declared that no competing interests exist.” Further, as requested, we also confirm that the correct funding sources are listed under “financial information”. Our updated “financial disclosure” statement should now read:
“This research was supported by a Social Sciences and Humanities Research Council of Canada grant to A.S.B at the University of British Columbia (# 435-2013-0286 and #895-2016-2011). The funders had no role in study design, data collection and analysis, decision to publish, or preparation of the manuscript. No authors received salary directly from the funding agencies. The authors have declared that no competing interests exist.”

Thank you for your help and patience,

Katharina Block (on behalf of Andrew Baron, Antonya Gonzalez, Clement Choi, Zoey Wong, and Toni Schmader).

**Reviewer 1**

1. **Semi-random assignment of condition: The authors note that “Each participant was semi-randomly assigned to one of three conditions…” Presumably due to the fact that the baseline data was collected at a different time, the authors describe the design as semi-random. I would recommend elaborating on the implications of the lack of full random assignment as a limitation in the discussion.**

   **Author Response:**
   Thank you for making this suggestion. We want to be absolutely transparent, so we included additional discussion of this design feature in the discussion section of our paper. In the discussion, we have now inserted the following on page 21

   “One limitation of our design is that data in the neutral condition was collected a few months after the data of the stereotypical and counter-stereotypical condition. While we took great care to collect the neutral condition with an age- and gender-matched sample, in the same setting and with the same research assistants, this non-random assignment to the control condition raises a small possibility of non-experimental effects. While significant differences between the concurrently collected conditions are significant and demonstrate effects of exposure to exemplars, differences from baseline could be somewhat more tentative. A replication study could demonstrate differences from baseline with greater certainty.”
2. **Self-concept and math: The authors report no differences by condition for self-concept (implicit or explicit) with math. The results do indicate that both girls and boys implicitly and explicitly associated themselves with math at similar rates. It may be helpful to address the lack of gender difference in math identity within the discussion, especially given that this finding contrasts with the findings on gender/math stereotypes.

   Author Response:**
   Thank you for pointing out that we could have paid more attention to this finding. To give more attention to the issue, we have now added the following to the discussion on page 24:

   ‘It is worth noting that, overall, we did not find significant gender differences in either explicit or implicit math self-concept. In addition, we found that effects of condition on implicit and explicit math self-concepts were not different for boys vs. girls (see SOM for details). Whereas gender differences in math self-concept persist in adolescents and adults [52], recent studies have found mixed results on gender differences in math performance and self-concept, with some studies showing no gender difference or reversed gender differences (i.e., greater math self-concept in girls) in young children [e.g., 53, 54], especially in some cultural contexts [55]. This could either be a sign of a positive societal trend or simply mean that gender stereotypes about math are not internalized and applied to the self-concept until a later age. Our sample in particular could have somewhat obscured gender differences in math self-concept at baseline because all children were tested in a science museum that presumably disproportionately attracts parents and/or children with an interest in STEM fields. To better estimate true gender differences in math self-concept in children, a large fully representative sample would be more appropriate.
3. **Median split for age: I was concerned by the analytical decision to perform a median split on age because median splits typically lose statistical power and can lead to spurious effects (MacCallum, Zhang, Preacher, & Rucker, 2002). It is not clear whether this decision was made to be consistent with the age groups in Gonzalez et al. (2021) or for some other theoretical reason. I would recommend incorporating additional analyses including age difference as a continuous variable (perhaps in the SOM if the median split is theoretically justified in the main text). This would allow readers a fuller picture of whether there are age-related changes in malleability.

   Author Response:**
   We agree with the reviewer here that there were several different options of how to analyze age-related effects. For our planned analyses we reasoned that the best option would be to go with the precedent set by past research. Gonzalez and colleagues’ analyses suggested a qualitative difference in younger vs. older children’s responses to counter-stereotypical racial exemplars (2021). Thus, categorical analyses (younger vs. older children) seemed theoretically appropriate to us. We now more clearly state this in the introduction (p. 8), and the results (p. 18).

   However, we also agree with the reviewer that analyses with age as a continuous variable can also give the reader important information. We have now done these analyses and found that they yield extremely similar patterns. We include the following description of these analyses in the SI on page 14/15:

“Our key analyses examined whether effects of condition differed between younger vs. older children, treating age-group as a categorical variable. However, changes with age could also arguably be quantitative rather than qualitative, making it more sensible to analyze age as a continuous moderator of condition effects on implicit gender stereotypes. When we did such analyses, we found a very similar pattern to the key analyses reported in the main manuscript; a descriptive pattern suggestive of an interaction with no statistically significant results. More specifically, we conducted a regression analysis where implicit gender stereotypes were predicted from gender (as a control variable), age (standardized), condition, and the age by condition interaction. Since the condition has three levels, we dummy coded with the counter-stereotypical condition as a reference group (so that dummy codes quantify how the counter-stereotypical condition differed from the control and the stereotypical conditions). Neither interaction terms for counter-stereotypical vs. control, b = -.05, *SE* = .05, *t*(311) = -0.93, *p* = .351, nor the interaction term for counter-stereotypical vs. stereotypical, b = -.09, *SE* = .05, *t*(311) = -1.69, *p* = .092, reached statistical significance. However, the interaction between age and the dummy code comparing the counter-stereotypical condition vs. the stereotypical condition was marginally significant, suggesting that implicit gender stereotypes may differ more between these two conditions for younger compared to older children. These results parallel our main manuscript results when we divide children into categories of older vs. younger.”

4. **Race and gender stereotype learning: In the discussion the authors state “Consistent with the second possibility, gender is seen as more self-relevant than race by some adolescents [47]…” Because the authors have previously argued that age is likely important for acquisition of stereotype relevant information about social groups, I would question whether citing this study of multiracial adolescents would support their claim. If the authors choose to keep this citation, it would be valuable to specify the ages of the participants and note the limits of generalizing between the cited study and the current study’s sample populations.

   Author Response:**
   Thank you for pointing out this nuance we had overlooked. We now did an additional literature search and added a citation [48] that shows a similar pattern in childhood.
5. **Minor comments:**
   a. **On line 463 (p. 20) and line 513 (p. 22) and the same sentence is repeated: “However, given the repeated nature..”

      Author Response:**
      We apologize for this oversight. We have removed the second sentence.
   b. **Figure 4 would be stronger with error bars included.

      Author Response:**
      Thank you, we have not updated Fig 4.
   c. **There is a small redundancy on line 117 (p. 5) “…exposure to at least frequent exposure…”

      Author Response:**
      Thank you for pointing this out, we have fixed this grammatical awkwardness.
   d. **“However, little systematic research has actually evaluated whether brief exposure to stereotype-related content in stories causally increases or decreases implicit gender stereotypes.” (line 118, p. 5) This should be qualified to be “...increased or decreases implicit gender stereotypes in children.” There is plenty of research on similar topics in adults (e.g., Dasgupta & Asgari, 2004).

      Author Response:**
      We absolutely agree with this point and indeed this work with adults helped to inspire the focus of the studies in this manuscript. We have now changed this sentence to read “However, little systematic research has actually evaluated whether brief exposure to stereotype-related content in stories causally increases or decreases implicit gender stereotypes in children.”

**Review 2 Comments:**

**Introduction**

1. **I realized later in the paper that the self-concept question was meant to be somewhat exploratory, but it fits awkwardly into the introduction section. For instance, on p. 3, it is presented as a third research question alongside what later appear to be the chief research questions (effects of counteresterotypic stories; moderation by age); this does not suggest that it is a lower-tier or more exploratory question. On the other hand, when I got to that part on page 3, I was surprised; I felt like an interest in “self-concept” came out of nowhere. I would recommend setting up why self-concept would be a reasonable thing to measure, even briefly, on that first page. Then, if the authors wish to “demote” that research question relative to the first two, that could be made clear in the listing of the questions.**

   **Author Response:**
   Thank you for helping us improve the structure of the paper. We have now mentioned the secondary focus on self-concept on page 1 and have tried to make more clear throughout the introduction how self-concept fits into the study. (see also response to point 4)
2. **On p. 4, paragraph 2, the literature review focuses heavily on examples of research with young girls. Perhaps this paragraph should not set up the idea of “across the lifespan”, unless the authors are making the case that the immediate relationships with identification & achievement have downstream consequences later in life. Otherwise, I would extend the literature covered in this section. E.g., a research example of implicit stereotypes predicting grades in college students: Ramsey & Sekaquaptewa (2011)**

   **Author Response:**
   Upon re-reading the topic sentence of this paragraph, we agree with the reviewer that it did not fully match the rest of the paragraph. Since adult evidence is discussed in other paragraphs, we have here removed the phrase “across the lifespan” as the reviewer suggested.
3. **One of the central justifications for the work is never supported empirically: p. 4, line 114 “children’s books about girls and women who excel in STEM-related fields have become increasingly popular.” I’d like to see some examples (Rosie Revere, Engineer? Doc MacStuffins?), and I’d like some quantification of “increasingly popular.”

   Author Response:**
   Thank you for pointing out the imprecisions here. We have added the example of the popular children’s book on the famous woman programmer Ada Lovelace. We also removed the word “increasingly” because there was only anecdotal or unpublished data to support a recent increase. We have also now cited a popular New York Times article to underscore the point that popular media outlet tout children’s books as a way to combat stereotypes. We hope that this has made the state of evidence more precisely described.
1. **On p. 6, I’m not convinced that the first paragraph makes sense where it is. Work on novel stereotype acquisition does not seem to speak to what this paper is going to test, since it’s established that children of the age group you will be testing have already acquired these math-gender stereotypes. I think this work should be presented in a different framing and a different spot. In contrast, the support from citations 25 and 26 in the next paragraph is much more directly compelling evidence.**

   **Nevertheless, as I continued through that paragraph on p. 6, I thought that it would make sense to add research comparing the ease of acquiring gender vs. race-based stereotypes, as my understanding was that gender-based ones might be more robust, and thus more resistant. This was addressed on p. 8, but the only citation is a review (I believe Ruble, Martin, & Berenbaum, 2006), and I think the argument would be strengthened with more primary sources, especially since the comparison between race and gender stereotypes becomes a heavy focus in the discussion later on.

   Author response:**
   Thank you for this suggestion. We have considered several ways in which we could address this comment. It was important to us to present the breadth of available evidence on the malleability of implicit associations in children. Therefore, we maintain that it is important to first present evidence that exposure to counter-stereotypic information is in principle successful at changing children’s implicit associations. However, we agree with the reviewer that we should have brought more clarity to the issue. We have thus revised our language in these two paragraphs to be clearer and added an additional citation [Rhodes M, Gelman SA. A developmental examination of the conceptual structure of animal, artifact, and human social categories across two cultural contexts. Cogn Psychol [Internet]. 2009;59(3):244–74. Available from: http://dx.doi.org/10.1016/j.cogpsych.2009.05.001] to support our claim that more research was needed to see whether effects from research on racial attitudes would generalize to children’s gender stereotypes.
4. **Related to point 1,on p. 9, a lot is made of the fact that no one has experimentally tested the impact of changing stereotypes on math self-concepts. I thought, “what a huge gap, this should be emphasized more!”, but then this question is treated like an afterthought in the analysis and discussion. I recommend more coherence: perhaps this section could be tempered so that the vibe is less “we are the first to tackle this important question” and more “because this is understudied, it is not clear if we’d see those effects, maybe yes (CITE) and maybe no (CITE), and so we will examine this as well.”**

   **Author Response:**
   Thank you for your suggestion. As stated in our response to point 1, we want to make clear that the effects of our counter-stereotypical exemplars on self-concept were a secondary focus of the current study. This is the case because it was yet to be determined whether counter-stereotypical exemplars could even shift gender *stereotypes* in children and without clarity on the effects of stereotypes, we deemed it unsure that we would see an effect on self-concept. In other words, the effects on stereotypes (which were the key focus of our study) were a necessary condition to see changes in self-concepts from our perspective. However, if stereotype change took place, clear directional hypotheses about the effects on self-concept would make sense based on balanced identity theory. Thus, we thought the framing hypotheses on changes in self-concept as more “secondary” made most sense. However, we see that we have been very unclear here. We now use the language “secondary” consistently. Further, as advised by the reviewer, we have toned down this section on page 9 as to not oversell our focus.
5. **P. 11, line 254: clarify - parents reported education and income to the authors? If so, please use past tense. If this is general science center visitor data, it should be cited.**

   **Author Response:**
   Thank you for pointing out that we had not clearly described this. The demographic data does not come from the particular parents of the children in the sample. The data does, however, come from a survey of parental demographics in the particular science center where our data was collected. We now explain this properly on page 11.
6. **P. 15, is citation 44 a source of recommendations for children in particular, or in general? Could add some context for how these error rates/rejection rates compare to adults.**

   **Author Response:**
   The cited paper provides recommendations for exclusion criteria on a related measure in adults (the Brief IAT), but has been generally used to guide exclusions when using reaction time measures of implicit associations. We have added this clarification on page 16 of the manuscript. Unfortunately, there is no well-validated exclusion criteria for children, so we chose to use well-validated exclusion criteria for the adult IAT. The total exclusion rate of 13.6% (for all causes) is quite common for research with children, and we have added supporting citations on page 16.
7. **My biggest question in the results is why a median split was employed. I understand that Gonzalez et al. (2020) split children into age groups, but from my reading, they did not do a median split. I’d be more convinced by some kind of developmental reason for why 8.8 is a meaningful cutoff. Otherwise, what about a regression analysis, looking at dummy coded condition interacting with age? Especially since gender didn’t matter in prior analysis, wouldn’t necessarily need to worry about three way interactions with gender. A stronger rationale for the median split is needed.**

   **Author Response:**
   As mentioned in the above response to o reviewer 1, comment 3, we wanted to use age groups that have had qualitatively different results in other studies of implicit association change in children (e.g., Gonzalez et al., 2017, 2021) However, we have added continuous age analyses to the SI, and results are comparable.
8. **Another major concern I have with the paper is frequent mentions of “descriptive differences” – I don’t think these are necessary and they may imply significance where there is none. The authors might refer people to figures without adding this language.**

   **Author Response:**
   Thank you for this comment. We agree that it is important to tread carefully when examining descriptive differences. We have now removed interpretation of descriptive differences in all but one instance. We still want to highlight the descriptive patterns with age since these patterns are opposite of what previous research has found with race. We believe it is important for the reader to be alerted to the fact that our patterns run counter to the age patterns that were previously found with intervention on racial stereotypes. If the reviewers and editor still disagree with this after our justification, however, we can remove these as well.
9. **I’m fascinated that girls showed implicit math self-identification, and I wonder if this is typical for girls?**

   **Author Response:**
   Please see response to reviewer 1, comment 2
10. **Several interesting arguments were made in the discussion (e.g., the need to reduce stereotypic exemplars, not just increase counterstereotypic; the two reasons offered for why gender and race stereotypes may operate differently). On p. 22, I’d offer another possibility: the categories “math and reading” are more discrete than “good and bad,” perhaps also allowing for easier absorption of examples? This would be a distinction between stereotyping and prejudice more broadly, and might be worth looking into the literature for any developmental lit for that?**

    Author Response:
    Thank you for making this suggestion. This is certainly a really intriguing possibility, and we would love to see future work examine this. However, we are somewhat hesitant to include this possibility explicitly in the discussion because we had trouble finding citations that would support the claim adequately. Indeed, when examining the malleability of novel associations, Gonzalez and colleagues (2017) found changes in response to new information did *not* differ significantly between evaluative (attitudes) and non-evaluate associations. In addition, looking through the literature, we were unable to find any evidence that math vs. reading were more discrete than good vs. bad. Some might even argue that good and bad a diametrically opposed categories, making them maximally different. So, we are unsure that we should make this argument. While an intriguing possibility, we have thus decided to not include it in the discussion section. If we misunderstood the reviewer, we are, of course, open to include the argument once we understand it correctly.
11. **On p. 22, line 509, confusing sentence - the prior sentence seems like it is about persistence already; is there a missing word?

    Author Response:**
    We agree that this made the flow of the paragraph confusing, so we deleted this sentence.
12. **The discussion mostly focuses on the effects of single brief interventions, which makes sense because that is what was tested. But given the emphasis on the rise of counterstereotypic books, I’d like to see some discussion of what might happen if, say, a classroom read counterstereotypic books all year - can the authors draw from any field intervention work and make a prediction?**

    **Author Response:**
    Thank you for making this suggestion. We have now added discussion on page 23 that specifically speculates on possible long-term outcomes of repeated interventions. Thi section of the discussion now reads as follows:

    “Future work will also need to determine how long the changes in implicit gender stereotyping as a result of this brief intervention changes persist. Work on the effectiveness of online interventions on changing adult’s implicit race bias suggest that, though initially effective [22], effects of numerous interventions dissipate completely after 24 hours [23]. In contrast, recent evidence suggests that story-based counter-stereotypical exemplars still measurably reduced children’s implicit race biases an hour after the intervention [27]. Given how uncertain the durability of stereotype-change is in children, future work may want to investigate how long-lasting effects of a single brief manipulation like ours is on children’s implicit math-gender stereotypes.

    However, given the repeated nature of exposure to stereotypical content in the real world, statistically significant changes in response to our brief content could potentially be very meaningful. With longer and/or repeated exposure to stories that counter gender stereotypes, effects could become stronger and/or more lasting. For example, recent research suggests that exposure to counter-stereotypical content in television programs reduces children’s racial biases upto four weeks later [48]. If children own several counter-stereotypical story books, like the popular book about Ada Lovelace cited in the introduction, and read them repeatedly, it is possible that their effects of such repeated exposure could strengthen and solidify effects.”
13. **Minor issues:**
    a. **P. 4, line 98: Word choice: in your description of implicit stereotypes, “domain” doesn’t sound like an attribute to me. To me, attribute would = trait (e.g., “good at math”; something that describes the group in question); otherwise it’s just another concept I think.**

       **Author Response:**
       Thank you for this comment. We have thought this issue over carefully and we maintain the terms “attribute” and “domain” are commonly used in the literature on implicit stereotypes measured with an IAT. An attribute is anything that is associated with a target category on the IAT. Math is a domain that is attributed more to boys than to girls.
    b. **P. 7, line 172: typo: “known”**
       **Author Response:**
       Thank you for finding this, we have fixed it.

**----- End of reviewer comments ----**
