# Supplementary material for: Exposure to stereotype-relevant stories shapes children’s implicit gender stereotypes
Source: PLoS One. 2022 Aug 3;17(8):e0271396. doi: 10.1371/journal.pone.0271396 (PMC9348658; doi:10.1371/journal.pone.0271396)
Supplement: Attachment — Submitted filename: ResponsetoReviewers2.docx [file pone.0271396.s003.docx]

Dear Dr. Shook,

Thank you for your help and quick turn around on this paper. We really appreciate the opportunity to publish our work (PONE-D-21-23166R1) at PLOSone. We thank you and the reviewers for taking another look at our manuscript and helping us improve the manuscript before publication. As detailed below, we have carefully addressed comments made by the reviewers in both the main manuscript and the supplement. With these changes, we believe we were able to address all concerns raised by the reviewers.

There are no changes to the financial disclosure we have to report. We have also uploaded our protocols and de-identified data under https://osf.io/2h3xu/.

Thank you for your time,

Katharina Block (on behalf of Andrew Baron, Antonya Gonzalez, Clement Choi, Zoey Wong, and Toni Schmader)

**Editor Comments:**

The reviewers and I greatly appreciate how thoroughly you addressed their concerns and suggestions. The manuscript is much improved and makes a nice contribution to the literature. The reviewers have a few minor suggestions to strengthen the paper. In particular, please report all of the results from the regression analysis with age as a continuous variable in the supporting materials (see Reviewer 1) and elaborate on issues related to the age median split (see Reviewer 2). Once these points are addressed, I am happy to accept the manuscript. 

**Comments to the Author**

**1. If the authors have adequately addressed your comments raised in a previous round of review …?**

Reviewer #1: All comments have been addressed

Reviewer #2: All comments have been addressed

**2. Is the manuscript technically sound, and do the data support the conclusions?**

Reviewer #1: Yes

Reviewer #2: Yes

**3. Has the statistical analysis been performed appropriately and rigorously?**

Reviewer #1: Yes

Reviewer #2: Yes

**4. Have the authors made all data underlying the findings in their manuscript fully available?**

Reviewer #1: Yes

Reviewer #2: Yes

**5. Is the manuscript presented in an intelligible fashion and written in standard English?**

Reviewer #1: Yes

Reviewer #2: Yes

**6. Review Comments to the Author**

**Reviewer #1:**

A. The authors have done a nice job in addressing the reviewer comments. They have successfully incorporated discussion of the limitation of non-random assignment and have nicely discussed implications of all results, including those related to math self-concept.

   **Author Response:**
   *Thank you for your time and your help with improving these parts of the manuscript. We are happy to hear that you are satisfied by our improvements.*
B. I noticed some incongruencies in how the participant age X condition analyses are reported across the main-text and supplement. That made it difficult to compare the continuous results against the categorical results. My only outstanding recommendation would be for the authors to synchronize the statistical reporting across the main-text and supplement. Specifically, I recommend reporting the following additional results to the supplemental analysis:

   1. The main effect of age
   2. Simple slope analyses to elaborate on the interaction effect (e.g., +/-1 SD on age)

   **Author Response:**
   *Thank you for these suggestions. Upon reading these sections side by side, we agree that make the main manuscript and the supplement for congruent would help the reader compared the results. As such, we have added the requested analyses and tried to format the results more in in line with the main manuscript. You can now find these analyses on p. 14-15 of the supplement (in tracked changes version).*

**Reviewer #2:**

A. Thank you for the opportunity to review this paper – I was an original reviewer on the manuscript. The original strengths of the paper are retained. The authors addressed my concerns about the introduction, discussion, and method by adding citations, using more careful language, changing the framing of the implicit stereotype change question, and clarifying the demographic data source. The counterarguments presented in explaining why some of my suggestions were not incorporated make perfect sense, and I appreciate the thorough and thoughtful response to each suggestion.

   **Author Response:**
   *Thank you for your gracious response. We really appreciate the thought and time you have put into helping us making our manuscript better.*
B. I looked carefully at how the median split results compared to the regression results. I think the addition to p. 9, setting up age as a categorical vs. continuous construct, is useful for setting up that analytic choice. I still think it is a bit unusual to use median split to distinguish between qualitative categories – if the group had been slightly older on average, the argument would be that a 9.5 year old is qualitatively different than a 9.6 year old, but in this case, the argument is that a 8.9 year old is qualitatively different than an 8.8 year old. In other words, median splits produce groups deemed qualitatively different based on the particular age range of that particular sample, rather than based on broader developmental trajectories. I might make that clearer in the document when comparing this median split to Gonzalez’ (p. 19 of the tracked changes edition). I think including the regression outcomes in the SI is also a good addition to the paper.

   **Author Response:**
   *We want to thank you again for your original comments as they have markedly improved our manuscript. We are happy to see that our addition on page 9 feel like an improvement to you. We have now added additional detail comparing the media split to Gonzalez et al. (2017) and additionally added citations for several related papers with similar splits. As we argue, there is a possibility that there is indeed a qualitative difference between age groups. However, we do not mean to suggest we provide strong evidence for this (vs. continuous change) yet. We acknowledge that future research has to be done to actually determine where exactly the shift take place on average (e.g., is the shift between 8 and 9 or 8.5 to 9.5 on average). Thus, we a) added a more detailed mention of analyses with age as a continuous variable (p. 19 of tracked version), and b) mention in the discussion the nature of changes with age need to be better understood with future research (p. 22 of tracked document).*
C. I note some small corrections below.
   a. P. 8: the new sentence beginning with “Particularly” is a fragment.

      **Author Response:**
      *Thank you for noticing that this sentence was unclear. It now reads: “Particularly, gender stereotypes merit separate investigation because children’s understanding of gender and race show different developmental trajectories [28]”*
   b. P. 20, line 368, “important to examine” instead of “important examine”

      **Author Response:**
      *Thank you for noticing this mistake, we have fixed it. Additionally, we have the whole manuscript another round of proofreading.*
